# PIM1 mediates epithelial-mesenchymal transition by targeting Smads and c-Myc in the nucleus and potentiates clear-cell renal-cell carcinoma oncogenesis

**DOI:** 10.1038/s41419-018-0348-9

**Published:** 2018-02-22

**Authors:** Bin Zhao, Lei Liu, Jun Mao, Zhiwei Zhang, Qifei Wang, Quanlin Li

**Affiliations:** 1 Department of Urology, The First Affiliated Hospital of Dalian Medical University, No. 222 Zhongshan Road, Dalian, 116011 China; 2 The Key Laboratory of Tumour Stem Cell Research of Liaoning Province, Dalian Medical University, Dalian, 116044 China

## Abstract

Emerging evidence has shown that the PIM serine/threonine kinase family, including PIM1, PIM2 and PIM3, is associated with tumour progression towards metastasis. PIM1, an attractive molecular target, has been identified as a potential prognostic biomarker for haematological and epithelial malignancies. However, to date, the potential regulatory roles and molecular mechanisms by which PIM1 affects the development and progression of cancers, including clear-cell renal-cell carcinoma (ccRCC), remain largely unknown. Herein, we present the first evidence that PIM1 is aberrantly overexpressed in human ccRCC tissues and cell lines and positively correlated with human ccRCC progression. In our study, depletion of PIM1 attenuated ccRCC cell proliferation, colony formation, migration, invasion and angiogenesis, suggesting that PIM1 expression may be a cancer-promoting event in ccRCC. Mechanistically, we observed that PIM1 could interact with Smad2 or Smad3 in the nucleus and subsequently phosphorylate Smad2 and Smad3 to induce the expression of transcription factors, including ZEB1, ZEB2, Snail1, Snail2 and Twist, to promote epithelial-mesenchymal transition (EMT). In addition, PIM1-mediated phosphorylation of c-Myc activates the expression of the above transcription factors to synergistically promote EMT but does not activate Smads. Collectively, our results demonstrate that aberrant expression of PIM1 contributes to ccRCC development and progression. Moreover, our data reveal a potential molecular mechanism in which PIM1 mediates crosstalk between signalling pathways, including different Smad proteins and c-Myc, which target downstream transcription factors (ZEB1, ZEB2, Snail1, Snail2 and Twist) to trigger EMT. Together, our data suggest that PIM1 may be a potential therapeutic target for ccRCC patients.

## Introduction

Renal cell carcinoma (RCC) remains one of the most commonly diagnosed malignant neoplasms in humans, with 63,990 new cases and 14,400 deaths predicted for 2017 in the US, and the annual morbidity and mortality rates of RCC are constantly rising^1^. Clear-cell renal-cell carcinoma (ccRCC), which comprises ~70% of RCCs, mostly present as an organ-confined disease, and surgical resection of localised ccRCC generally leads to excellent long-term disease-free survival (DFS)^2,3^. However, advanced ccRCC has a poor survival rate and may result in metastasis or recurrence, which is predominantly attributed to resistance to both traditional chemotherapy and radiation, after the initial radical surgery^4,5^. Hence, it is of importance to understand the underlying molecular mechanisms of malignant ccRCC and identify new efficacious therapeutic strategies.

The PIM kinase family consists of three constitutively active members, namely, PIM1, PIM2 and PIM3, which encode serine/threonine kinases with a broad range of cellular substrates that have been identified as oncogenes in multiple human malignant solid tumours^6,7^. PIM1, an attractive gene target, was first identified in murine leukaemia virus (MuLV)-induced lymphoma models, and the oncogenic activity of PIM1 was subsequently discovered^8^. Previous evidence has shown that overexpression of PIM1 in various human cancers, such as breast cancer, mesothelioma and glioblastoma^9–11^, is well correlated with the processes of cancer progression, including cell proliferation, cell cycle arrest, apoptosis, migration, invasion and drug resistance. However, the expression profile and role of PIM1 in ccRCC remain unclear. In addition, PIM1 exerts its tumorigenicity by regulating c-Myc; PIM1 phosphorylates c-Myc at S62, which increases c-Myc protein stability, thereby enhancing the transcriptional activity of c-Myc^12^. PIM1 synergises significantly with c-Myc to promote the development of cancer^13^. These results prompted us to test whether the interaction between PIM1 and c-Myc is involved in ccRCC progression and metastasis.

Epithelial-mesenchymal transition (EMT), which comprises multiple dynamic transitional states between epithelial and mesenchymal phenotypes, plays an essential role in the regulation of cancer progression and metastasis^14^. EMT is characterised by several key events: cell polarity changes, including loss-of-apical-basal polarity and the establishment of front-rear polarity; reorganisation of the cytoskeleton; downregulation of the epithelial marker E-cadherin to disassemble cell junctions; upregulation of the mesenchymal markers N-cadherin and Vimentin to increase cell protrusions and motility; and degradation of the extracellular matrix (ECM) to acquire invasive properties. Furthermore, EMT is orchestrated and highly modulated by a number of upstream transcription factors, such as ZEB1, ZEB2, Snail1, Snail2 and Twist, and other regulators and processes, including non-coding miRNAs and alternative splicing^15–21^. Remarkably, the TGF-β signalling pathway, a crucial driver of EMT, is associated with cancer progression, migration, invasion and dissemination^22^. The Smad protein family, including Smads 1–7, plays a pivotal role in the TGF-β signalling pathway^23^. Despite the importance of TGF-β-induced EMT as a regulator of tumourigenesis, the mechanisms underlying EMT in ccRCC have not been characterised in detail.

In this study, we showed that PIM1 expression is elevated in human ccRCC tissues and cells and that PIM1 expression is positively correlated with human ccRCC progression. A series of in vitro and in vivo experiments indicated that stable knockdown of PIM1 expression in ccRCC cells significantly impaired their capacity for proliferation, migration, invasion and angiogenesis. Additionally, our study identified an underlying molecular mechanism in which PIM1 regulates ccRCC development and progression by mediating crosstalk between signalling pathways, including independent Smad proteins and c-Myc, which target downstream transcription factors (ZEB1, ZEB2, Snail1, Snail2 and Twist) to trigger EMT.

## Results

### PIM1 is aberrantly overexpressed in ccRCC and positively correlated with ccRCC progression

**Fig. 1:**
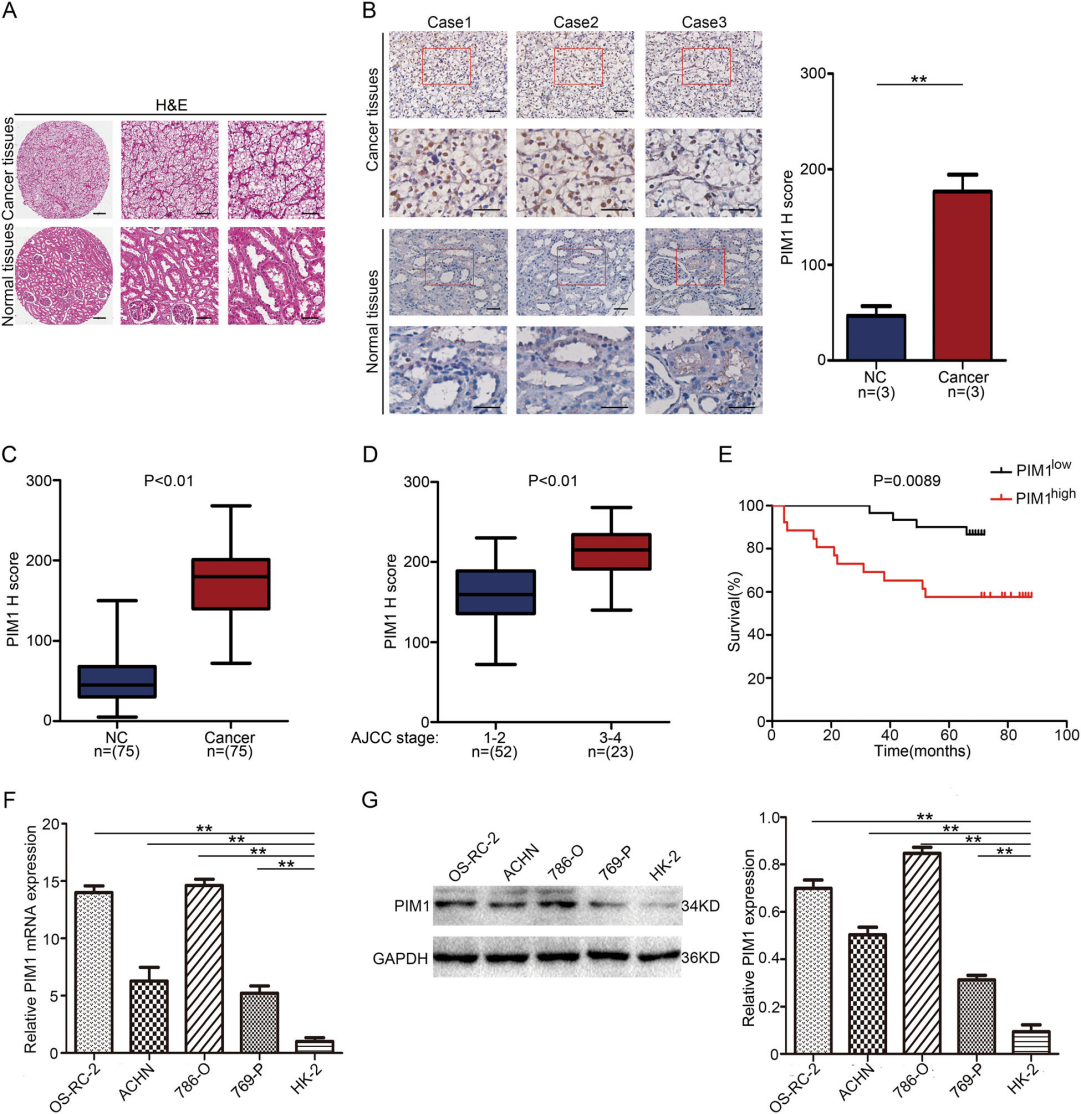
Aberrant overexpression of PIM1 in human ccRCC. **a** Representative image of haematoxylin and eosin staining (H&E) of the ccRCC tissue microarray. Scale bars: 200 μm, 100 μm and 50 μm, respectively. **b** Representative cases of immunohistochemistry (IHC) for PIM1 expression in the ccRCC tissue microarray. Scale bars: 20 μm and 10 μm, respectively. PIM1 IHC staining scores in the carcinoma tissues (*n* = 3) and surrounding tissues (*n* = 3) are shown on the right. Data are shown as the mean ± S.E.M. of the values from triplicate experiments. Statistical significance was determined with a two-tailed Student’s *t*-test. **P < 0.01. **c**, **d** PIM1 IHC staining scores in the carcinoma tissues (*n* = 75) and adjacent tissues (**c**) (*n* = 75) or in the cancer tissues (*n* = 75) in different AJCC stages (**d**). NC represents non-cancerous tissue. AJCC stages: I–IV. The horizontal lines in the box plots show the median, the boxes show the interquartile range, and the whiskers show the 2.5th and 97.5th percentiles (Wilcoxon signed-rank test or Mann–Whitney *U*-test). **e** Survival curve of the kidney cancer patients with high (>median) H-scores (*n* = 30) or low (<median) H-scores (*n* = 26). Statistical significance was determined with the log rank test (*p* = 0.0089). **f** qRT-PCR analysis of the relative PIM1 mRNA expression level in a panel of four human ccRCC cell lines and an immortalised proximal tubule epithelial cell line, HK-2. The relative PIM1 mRNA expression level was normalised to GAPDH. Data are shown as the mean ± S.E.M. of the values from three independent experiments. Statistical significance was determined with a two-tailed Student’s *t*-test. **P < 0.01. **g** Immunoblotting analysis of PIM1 expression in ccRCC cells and HK-2 cells. GAPDH was used as the loading control, and the PIM1 protein levels in the above cell lines were quantified by densitometry. Data are shown as the mean ± S.E.M. of the values from three biological replicates. Statistical significance was determined with a two-tailed Student’s *t*-test. **P < 0.01

To investigate the role of PIM1 in human ccRCC, PIM1 expression was first analysed in a tissue microarray of ccRCC containing cancer samples and adjacent non-cancer samples. Representative haematoxylin and eosin (H&E) staining for the ccRCC tissue microarray and representative cases of immunohistochemistry (IHC) for PIM1 expression in the tissue microarray are shown (Fig. 1a, b). We found that the PIM1 protein was expressed in the nucleus and that its levels were significantly higher in carcinoma tissues than in the surrounding normal tissues (Fig. 1c). Further analysis showed that PIM1 protein levels were markedly higher in ccRCC tissues at the late American Joint Committee on Cancer (AJCC) stages than in ccRCC tissues at the AJCC early stages (Fig. 1d). In addition, survival curve analysis revealed that patients with high PIM1 expression (>median H-scores) had a dramatically lower survival rate than patients with low-PIM1 expression (<median H-scores) (Fig. 1e). The median survival time of kidney cancer patients with high-PIM1 expression was significantly shorter than that of those with low-PIM1 expression. To further test PIM1 overexpression in kidney cancer, we utilised a panel of four human ccRCC cell lines (ACHN, 786-O, 769-P and OS-RC-2) and HK-2 cells (an immortalised proximal tubule epithelial cell line) to examine relative PIM1 mRNA expression by qRT-PCR. The results indicated that PIM1 was overexpressed in ccRCC cell lines (Fig. 1f), which was consistent with the results of the immunoblotting assay (Fig. 1g). Taken together, these results clearly suggest that PIM1 is aberrantly overexpressed in human ccRCC tissues and cell lines and positively correlated with human kidney cancer progression.

### PIM1 promotes the growth of ccRCC cells in vitro

**Fig. 2:**
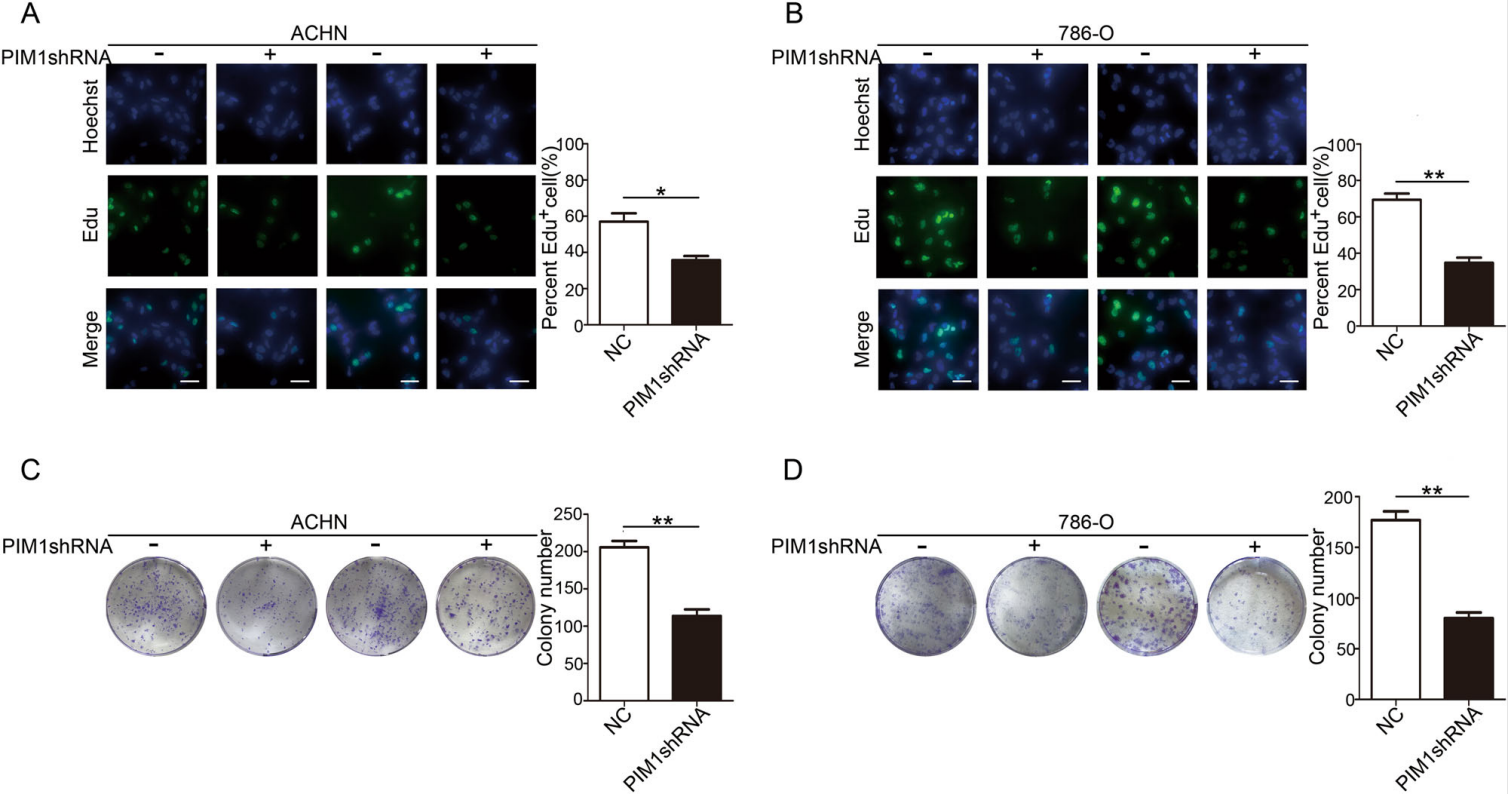
Knockdown of PIM1 suppresses the growth of ccRCC cells in vitro. **a**, **b** Edu assays. The cells were stained with Edu (green) and a nuclear dye, Hoechst (blue). Edu-positive cells were counted with ImageJ software, and the percentage of positive cells was calculated. Scale bar: 20 μm. **c**, **d** Colony formation assays. The number of colonies were quantified with ImageJ software. Data are shown as the mean ± S.E.M. of the values from triplicate experiments. Statistical significance was assessed with a two-tailed Student’s *t*-test. *P < 0.05; and **P < 0.01

To verify that PIM1 overexpression promotes kidney cancer, we generated four ccRCC cell lines using both ACHN and 786-O cells to measure proliferation in vitro. As shown by the Edu assay results, depletion of PIM1 with the lentiviral shRNA interference technique dramatically attenuated the number of Edu-positive ACHN and 786-O cells, suggesting that silencing of PIM1 effectively inhibits cell proliferation in vitro (Fig. 2a, b). To examine whether knockdown of PIM1 could affect the long-term proliferation of ccRCC cells, the cells were used in colony formation assays for the indicated interval. We observed that the ability of ACHN and 786-O cells to form colonies was remarkably impaired when PIM1 was knocked down (Fig. 2c, d). Collectively, these data demonstrate that PIM1 overexpression in ccRCC cells may be a cancer-promoting event in ccRCC.

### Depletion of PIM1 suppresses the migration, invasion and angiogenesis behaviours of ccRCC cells in vitro

**Fig. 3:**
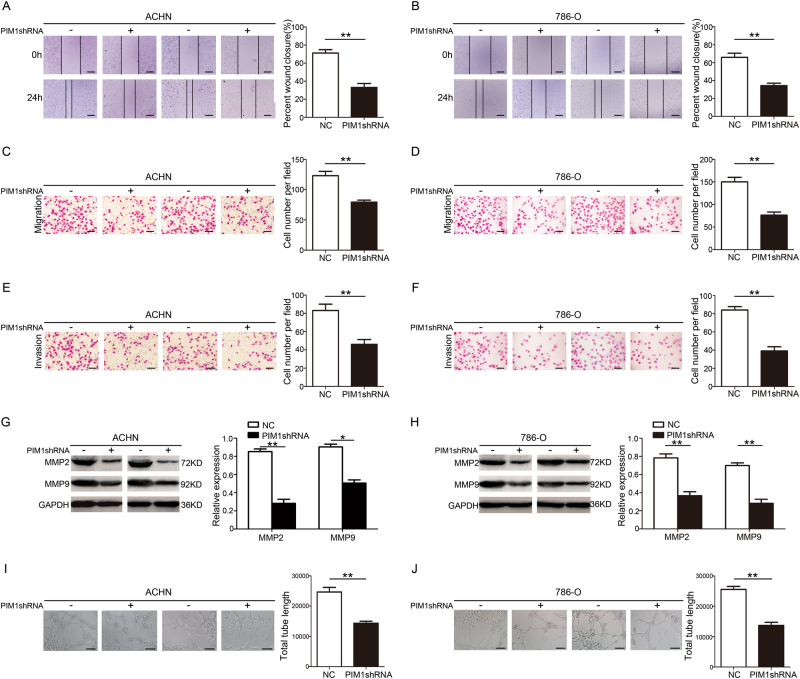
Depletion of PIM1 inhibits the migration, invasion and angiogenesis behaviours of ccRCC cells in vitro. **a**, **b** Wound-healing assays. The wound closure rate was calculated. Scale bar: 200 μm. **c**, **d** Transwell migration assays. The cell number per field was calculated. Scale bar: 20 μm. **e**, **f** Matrigel Transwell assays. The cell number per field was determined. Scale bar: 20 μm. **g**, **h** Immunoblotting analysis of MMP2 and MMP9 protein levels. GAPDH was used as the loading control, and the indicated proteins were quantified with ImageJ software. **i**, **j** Capillary tube formation (CTF) assays. The total length of tubes formed by HUVECs was calculated with ImageJ software. Scale bar: 50 μm. All data represent the mean ± S.E.M. of the values from three biological replicates. Statistical significance was determined with a two-tailed Student’s *t*-test. *P < 0.05; and **P < 0.01

Having observed a dramatic change in cell proliferation following the depletion of PIM1, we utilised a series of assays to detect whether knockdown of PIM1 affected ccRCC cell migration, invasion and angiogenesis. Wound-healing assays revealed that PIM1-depleated ACHN and 786-O cells migrated significantly less than cells transfected with control-shRNA (Fig. 3a, b), which was also observed in Transwell migration assays (Fig. 3c, d). Next, Matrigel Transwell assays showed that PIM1 depletion greatly inhibited the invasion capacity of ACHN and 786-O cells (Fig. 3e, f). A previous study showed that degradation of extracellular matrix (ECM) by matrix metalloproteinases potentially contributes to cancer progression, invasion and metastasis^24^. Immunoblotting assays showed that PIM1 silencing clearly decreased MMP2 and MMP9 expression (Fig. 3g, h). Intriguingly, a recent study showed that angiogenesis is positively correlated with tumourigenesis and potentially promotes tumour growth and metastasis^25^. Capillary tube formation (CTF) assays indicated that depletion of PIM1 strongly blocked angiogenesis (Fig. 3i, j). Collectively, these data reveal that PIM1 potentially contributes to ccRCC tumour migration, invasion and angiogenesis in vitro.

### Depletion of PIM1 blocks EMT in ccRCC cells

**Fig. 4:**
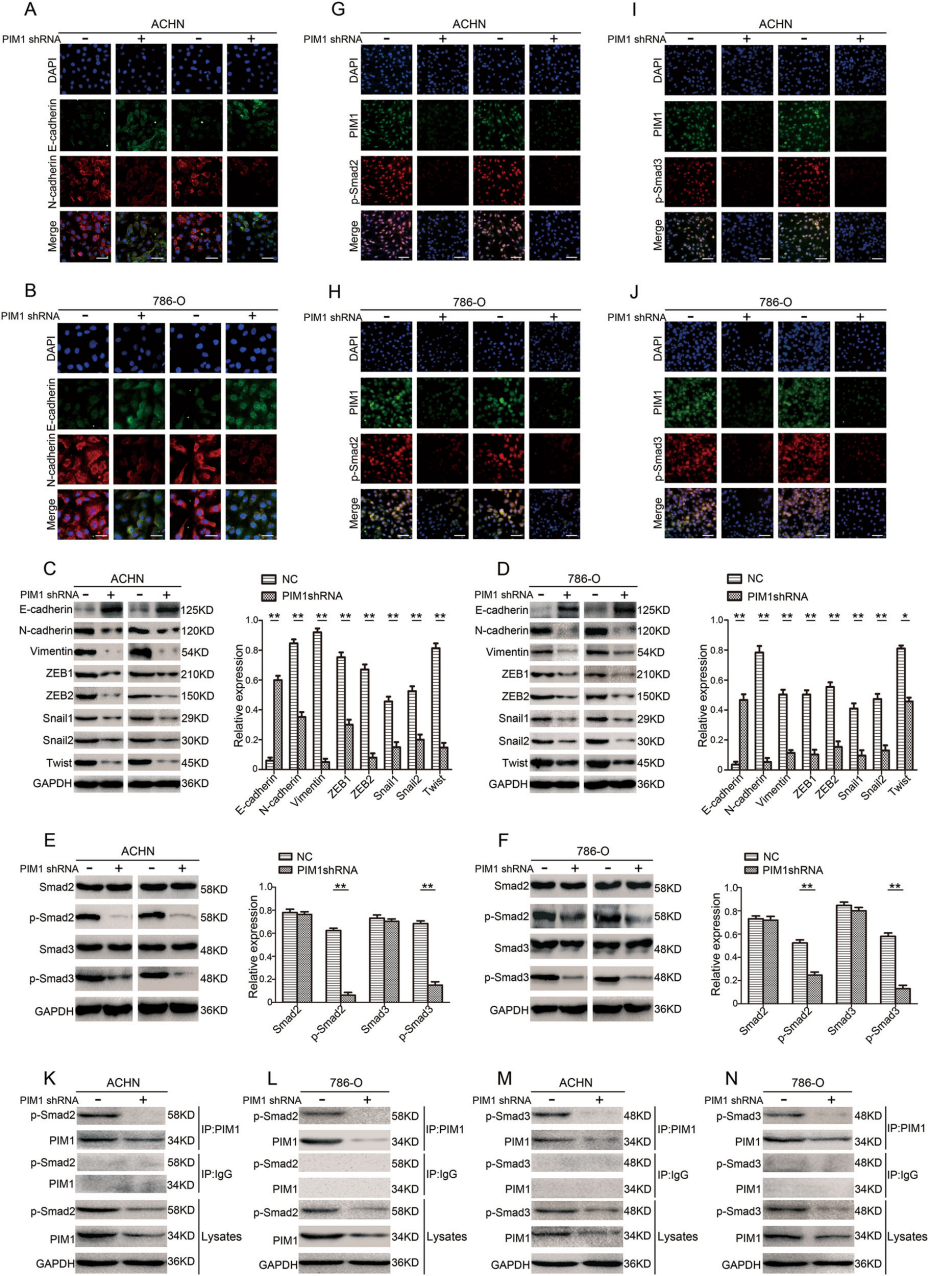
Depletion of PIM1 blocks EMT in ccRCC cells by targeting Smads. **a**–**d** Knockdown of PIM1 suppresses EMT in ACHN and 786-O cells. **a**, **b** Immunofluorescence staining for E-cadherin and N-cadherin. Panels show representative images from one of three experiments. Scale bar: 20 μm. **c**, **d** Immunoblotting analysis of E-cadherin, N-cadherin, Vimentin, ZEB1, ZEB2, Snail1, Snail2 (Slug) and Twist protein levels. GAPDH was used as the loading control, and the levels of the above proteins were quantified with ImageJ software. **e**–**n** PIM1 interacts with Smad2 or Smad3 in the nucleus and then phosphorylates Smad2 and Smad3. **e**, **f** Immunoblotting analysis of the Smad2, Smad3, p-Smad2 (S467) and p-Smad3 (S423 and S425) protein levels. GAPDH was used as the loading control, and the levels of the above proteins were quantified with ImageJ software. **g**–**j** Co-localization immunofluorescence analysis for PIM1, p-Smad2 and p-Smad3. Panels show representative images from one of three experiments. Scale bar: 50 μm. **k**–**n** Co-immunoprecipitation (co-IP) assays. Endogenous PIM1 was immunoprecipitated, and the eluted proteins were probed for p-Smad2 or p-Smad3. Lysates were also subjected to co-IP with an IgG control. All data represent the mean ± S.E.M. of the values from three independent experiments. Statistical significance was determined with a two-tailed Student’s *t*-test. *P < 0.05; and **P < 0.01

To determine the underlying molecular signalling pathways through which depletion of PIM1 elicits anti-proliferation, anti-migration and anti-invasion effects on cancer cells, we examined the effects of PIM1 depletion on EMT in ccRCC cells. Immunofluorescence assays showed dramatic upregulation of the epithelial phenotype marker E-cadherin and downregulation of the mesenchymal phenotype marker N-cadherin in both ACHN and 786-O cells transfected with PIM1shRNA or control-shRNA (Fig. 4a, b). Furthermore, immunoblotting assays showed that knockdown of PIM1 significantly increased E-cadherin protein expression levels and decreased N-cadherin and Vimentin protein expression levels (Fig. 4c, d). Next, we performed immunoblotting assays to analyse upstream transcription factors that are associated with EMT, including ZEB1, ZEB2, Snail1, Snail2 and Twist proteins. The results showed that silencing of PIM1 markedly inhibited the expression of the above transcription factors compared with control cells (Fig. 4c, d). Collectively, these data reveal that depletion of PIM1 attenuates EMT in ccRCC cells via downregulation of the above transcription factors.

### PIM1 regulates Smads via interactions with Smad2 or Smad3 in the nucleus, subsequently phosphorylating Smad2 and Smad3 to induce EMT

To investigate the molecular mechanism underlying EMT in ccRCC cells, immunoblotting assays were performed to quantify the expression levels of Smad2, Smad3, p-Smad2 and p-Smad3 in both ACHN and 786-O cells transfected with PIM1shRNA or control-shRNA. Intriguingly, we found that expression levels of total Smad2 and Smad3 proteins were not notably changed in the PIM1-depleated ccRCC cells. However, depletion of PIM1 strongly reduced the levels of phosphorylated Smad2 and Smad3 (Fig. 4e, f). These findings in ACHN and 786-O cells raised the possibility that PIM1 regulates Smads by mediating the phosphorylation of Smad2 and Smad3 in the nucleus, thereby inducing EMT. To test this hypothesis, co-localization immunofluorescence assays were performed using ACHN and 786-O cells to determine whether PIM1 interacts with p-Smad2 or p-Smad3 in the nucleus. The results clearly showed interactions between endogenous PIM1 and endogenous p-Smad2 in the nuclei of both ACHN and 786-O cells, whereas knockdown of PIM1 remarkably attenuated the interaction between PIM1 and p-Smad2 in the nucleus (Fig. 4g, h). A similar interaction pattern was observed between PIM1 and p-Smad3 in both ACHN and 786-O cells (Fig. 4i, j). Next, co-immunoprecipitation (co-IP) assays with a PIM1-specific antibody revealed that p-Smad2 co-immunoprecipitated with PIM1 and that the interaction between endogenous PIM1 and endogenous p-Smad2 was significantly decreased following knockdown of PIM1 (Fig. 4k, l). Similarly, p-Smad3 was also precipitated with the anti-PIM1 antibody in ACHN and 786-O cells (Fig. 4m, n). Additionally, an in vitro PIM1 kinase assay revealed that PIM1 directly phosphorylated Smad2 and Smad3 and that inhibition of PIM1 kinase activity with SGI-1776 significantly reduced the protein levels of p-Smad2 and p-Smad3 (Supplementary Figure 1A and B). Notably, the co-IP assays indicated that Smad2 did not interact with Smad3 in the PIM1/Smad complex, suggesting that the PIM1/Smad complex is either a PIM1/Smad2 complex or a PIM1/Smad3 complex (Supplementary Figure 2A and B). Together, these results show that PIM1 can interact with Smad2 or Smad3 in the nucleus and subsequently phosphorylate Smad2 and Smad3 to induce EMT.

### The interaction between PIM1 and c-Myc triggers EMT but does not activate Smads

**Fig. 5:**
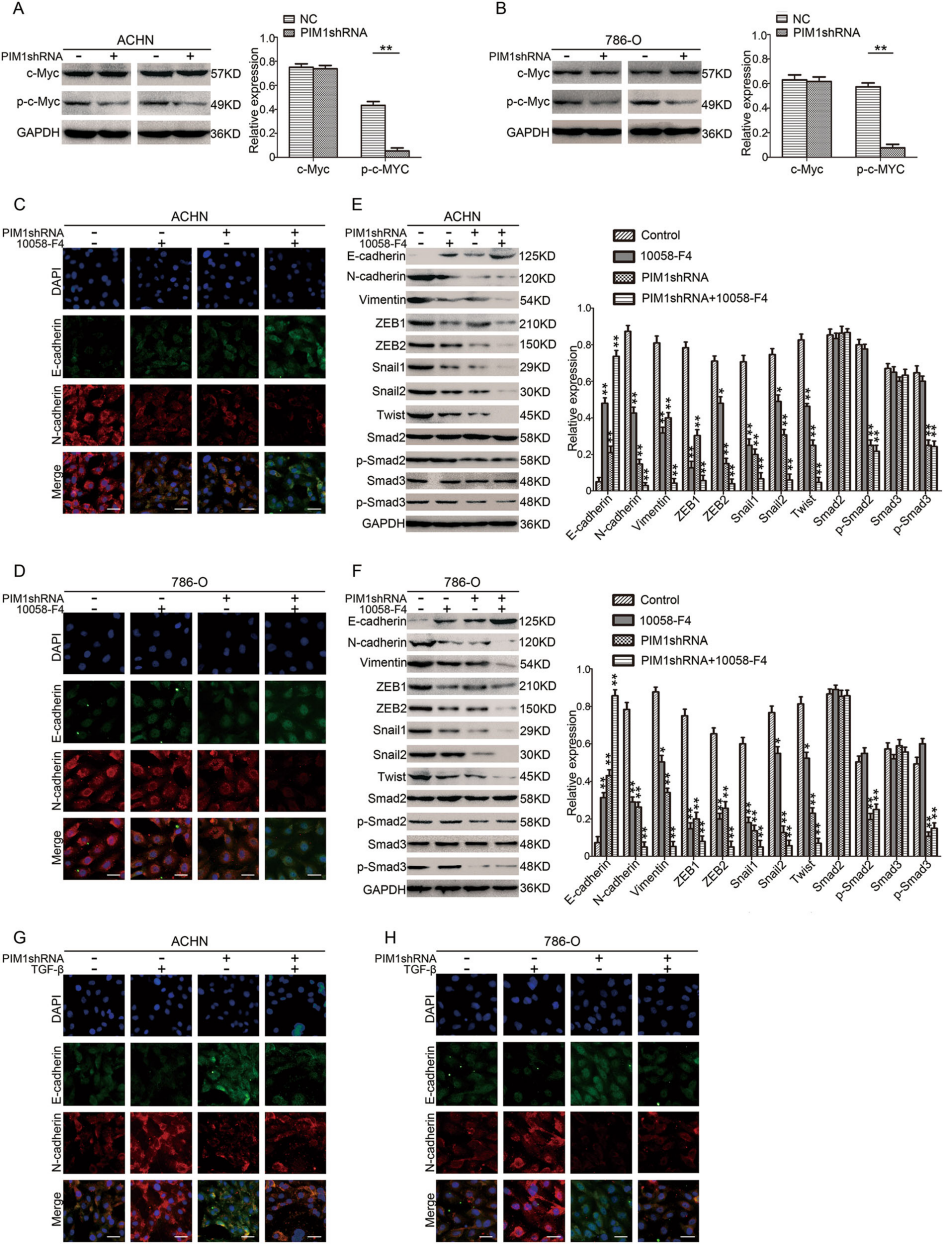
Interaction between PIM1 and c-Myc triggers EMT in ccRCC cells but does not activate Smads. **a**, **b** Immunoblotting analysis of c-Myc and p-c-Myc (S62) protein levels. GAPDH was used as the loading control, and the levels of the above proteins were quantified with ImageJ software. **c**, **d** Immunofluorescence assays. Cells were treated with or without 40 µM 10058-F4 for 24 h. After the 24-h treatment, cells were fixed and stained with antibodies against E-cadherin and N-cadherin and DAPI. The panels show representative images from one of three experiments. Scale bar: 20 μm. **e**, **f** Immunoblotting assays. Cells were treated with or without 10058-F4 for 24 h. After the 24-h treatment, proteins were extracted. Immunoblotting assays were performed to analyse E-cadherin, N-cadherin, Vimentin, ZEB1, ZEB2, Snail1, Snail2 (Slug), Twist, Smad2, Smad3, p-Smad2 (S467) and p-Smad3 (S423 and S425) protein levels. GAPDH was used as the loading control, and the levels of the above proteins were quantified with ImageJ software. **g**, **h** Immunofluorescence assay. Cells were treated with or without 10 ng/mL TGF-β for 24 h. After the 24-h treatment, cells were fixed and stained with antibodies against E-cadherin and N-cadherin and DAPI. The panels show representative images from one of three independent experiments. Scale bar: 20 μm. All data represent the mean ± S.E.M. of the values from three experiments. Statistical significance was determined with a two-tailed Student’s *t*-test or one-way ANOVA. *P < 0.05; and **P < 0.01

We next investigated whether c-Myc participates in the PIM1-mediated activation of EMT. Immunoblotting and co-IP assays were performed, and the results indicated that PIM1 interacts with c-Myc and phosphorylates c-Myc at S62 moreover, knockdown of PIM1 strongly decreased the level of p-c-Myc but not total c-Myc (Fig. 5a, b and Supplementary Figure 3A and B). We also utilised a specific inhibitor (10058-F4) that targets both c-Myc and p-c-Myc to detect the role of c-Myc in the regulation of EMT by PIM1. Immunofluorescence assays showed that 10058-F4 treatment further upregulated E-cadherin expression and downregulated N-cadherin expression (Fig. 5c, d). Furthermore, immunoblotting assays revealed that the presence of 10058-F4 significantly increased E-cadherin protein levels and repressed N-cadherin and Vimentin expression levels (Fig. 5e, f). Additionally, 10058-F4 treatment remarkably reduced the expression of transcription factors ZEB1, ZEB2, Snail1, Snail2 and Twist (Fig. 5e, f). However, 10058-F4 treatment had no obvious effect on Smad proteins (Fig. 5e, f). We thereby speculated that phosphorylation of c-Myc via interactions with PIM1 activates the expression of the above transcription factors to promote EMT without activating Smads. Next, we stimulated the TGF-β signalling pathway with TGF-β to verify our speculation. Immunofluorescence assays showed that depletion of PIM1 blocked EMT by inhibiting crosstalk between signalling pathways, including independent Smads and c-Myc, and that this process was reversed by TGF-β treatment, which led to the downregulation of E-cadherin expression and upregulation of N-cadherin expression compared to untreated cells (Fig. 5g, h). Taken together, our data suggest that phosphorylation of c-Myc via interactions with PIM1 activates the expression of downstream transcription factors (ZEB1, ZEB2, Snail1, Snail2 and Twist), without activating Smads, to synergistically promote EMT.

### Oncogenic activity of PIM1 in vivo

**Fig. 6:**
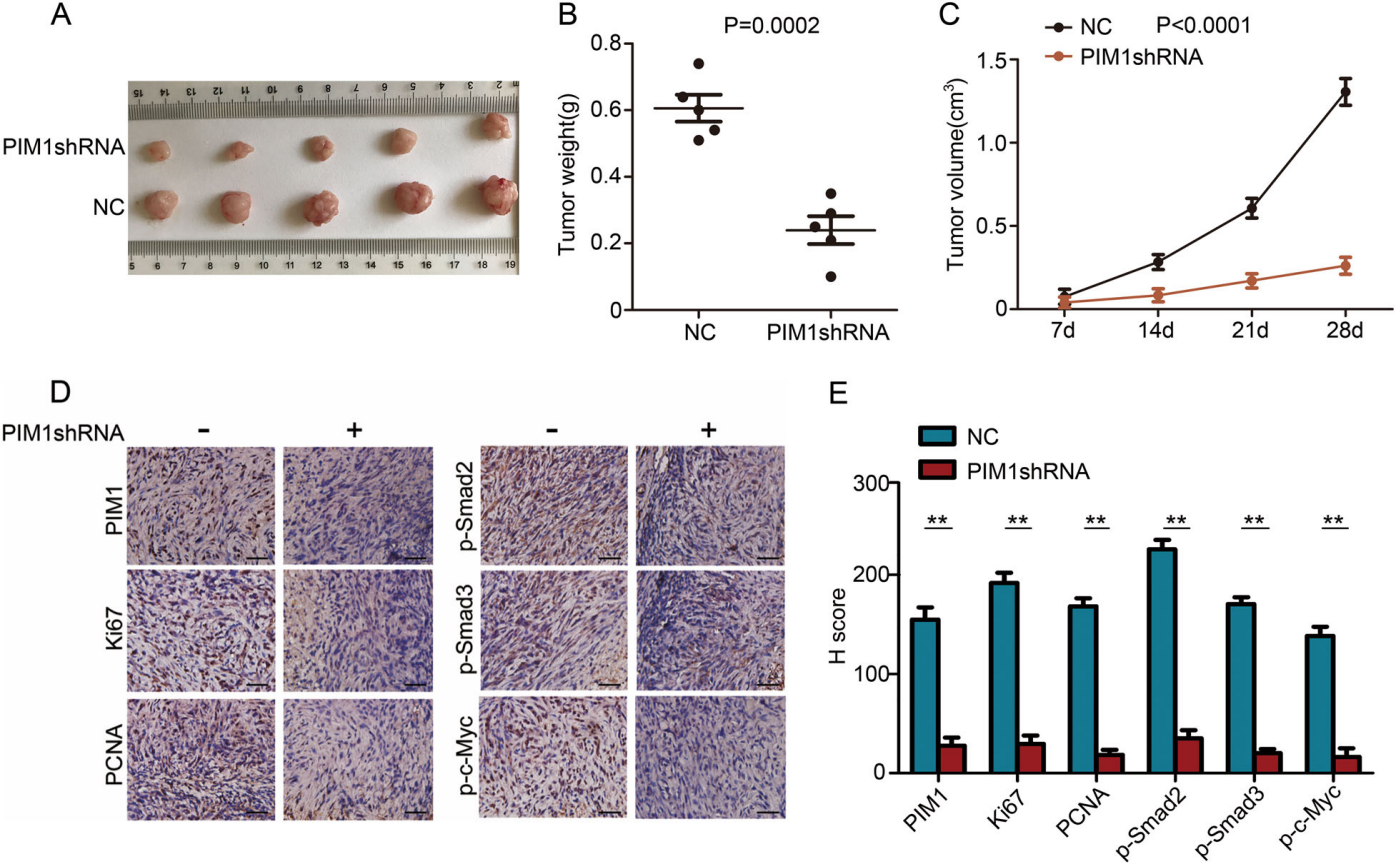
Tumorigenicity of PIM1 in vivo. **a** Following subcutaneous injections of ACHN cells (PIM1shRNA/NC) in athymic nude mice (*n* = 5/per group) and tumour growth for 28 days, photographs of the tumours were obtained at necropsy. **b** Mice were killed 28 days after the subcutaneous injection. Scatter plot analysis of the mouse tumour weights. The data are shown as the mean ± S.E.M., and statistical significance was determined with a two-tailed Student’s *t*-test (*P* = 0.0002). **c** The volumes of the generated tumours were measured weekly. The data are shown as the mean ± S.E.M., and statistical significance was determined with a two-tailed Student’s *t*-test (*P* < 0.0001). **d** IHC for PIM1, Ki67 and PCNA in the tumours from each group. The tumour edge tissues of the PIM1-depleted and control groups were subjected to IHC for p-Smad2, p-Smad3 and p-c-Myc. Scale bar: 20 μm. **e** The IHC staining scores for PIM1, Ki67, PCNA, p-Smad2, p-Smad3 and p-c-Myc in the carcinoma tissues. The data are shown as the mean ± S.E.M. of the values from triplicate experiments. Statistical significance was assessed with a two-tailed Student’s *t*-test. **P < 0.01

To further investigate the role of PIM1 in ccRCC cell growth in vivo, an athymic nude mouse model was examined. Photographs of the tumours at necropsy and the measured volumes of the tumours indicated that PIM1-depleated ACHN cells grew much more slowly than control cells (Fig. 6a, c). The weights of the tumours from these mice were remarkably lower than those from the control mice (Fig. 6b). IHC was performed to further quantify the degree of PIM1 depletion in the tumour tissues. The results showed that PIM1 expression in the PIM1shRNA group was significantly decreased compared with that in the control group (Fig. 6d, e). Furthermore, IHC analysis of the proliferative markers Ki67 and PCNA in tumours from each group revealed that there was limited expression of Ki67 and PCNA in the PIM1-deficient tumour tissues, which indicated slower growth compared to the control tissues (Fig. 6d, e). In addition, IHC for p-Smad2, p-Smad3 and p-c-Myc was performed on the tumour edge tissues of the PIM1 knockdown mice and control mice to confirm tumour invasiveness. The results revealed that the expression levels of p-Smad2, p-Smad3 and p-c-Myc in the PIM1-deficient tumour edge tissues were significantly lower than those in the control tumour edge tissues (Fig. 6d, e). Collectively, these results demonstrate that PIM1 functions as a tumour promoter in ccRCC and that depletion of PIM1 dramatically attenuates ccRCC oncogenic properties in vivo.

## Discussion

**Fig. 7:**
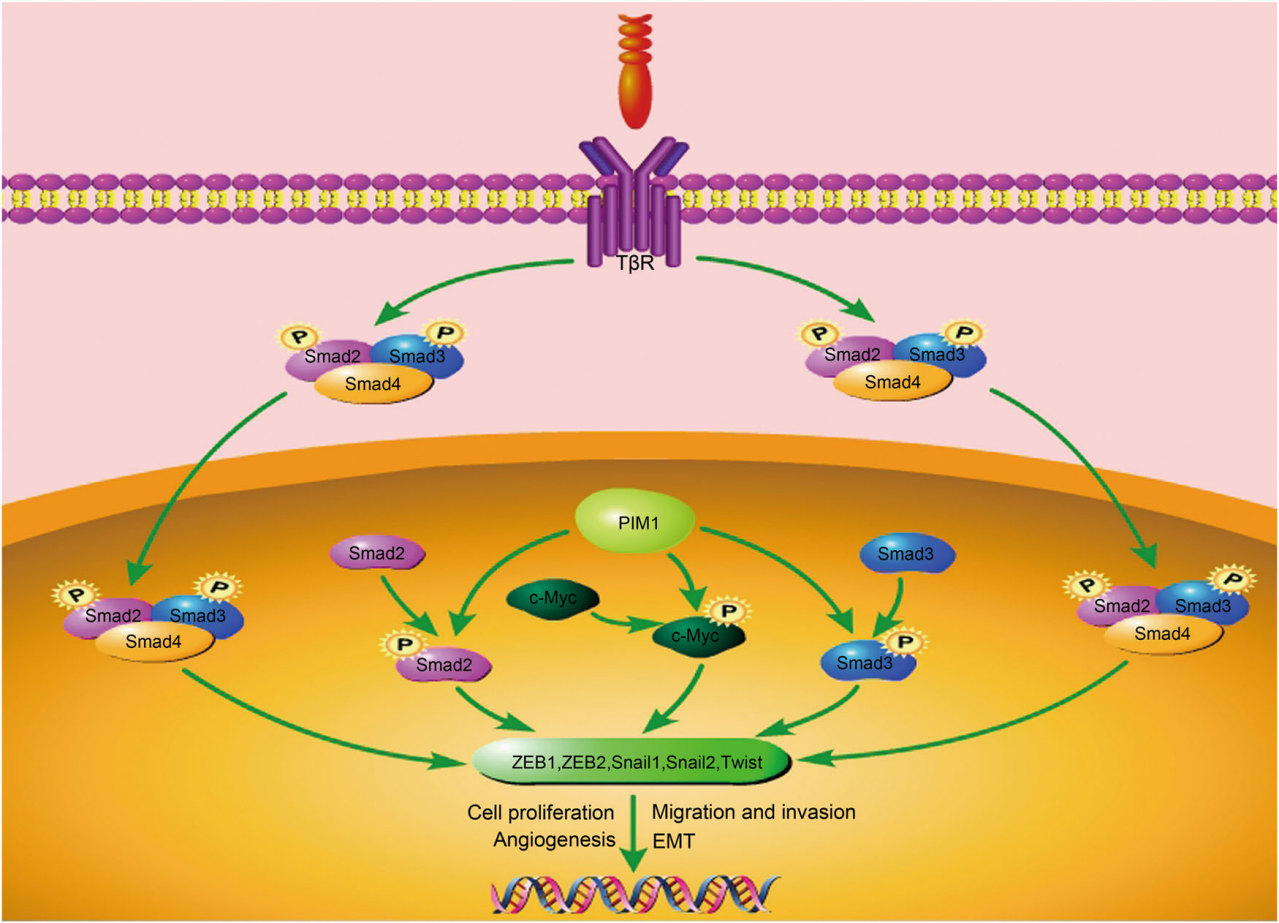
Schematic representation of the potential function and mechanism of PIM1 in human ccRCC

In this study, we demonstrated the roles of PIM1 in human ccRCC and determined the possible underlying mechanism. We showed that PIM1 was aberrantly overexpressed in human ccRCC tissues and cell lines and positively correlated with ccRCC progression. Furthermore, PIM1 promoted the growth of ccRCC tumours in vivo and tumourigenesis in vitro. Depletion of PIM1 suppressed the migration, invasion and angiogenesis behaviours of ccRCC cells in vitro, indicating that PIM1 may be a cancer-promoting protein in kidney cancer. We further showed that PIM1 could interact with and phosphorylate Smad2 or Smad3 in the nucleus to induce transcription factor (ZEB1, ZEB2, Snail1, Snail2 and Twist) expression and EMT. In addition, phosphorylation of c-Myc through its interaction with PIM1 activated the expression of the above transcription factors to synergistically promote EMT without activating Smads. These findings revealed a potential molecular mechanism in which PIM1 mediates crosstalk between signalling pathways, including independent Smads and c-Myc, which then target downstream transcription factors (ZEB1, ZEB2, Snail1, Snail2 and Twist) to trigger EMT; moreover, hyperactivation of EMT was shown to be associated with ccRCC cell proliferation, motility and invasion, as well as angiogenesis (Fig. 7).

In this study, PIM1 was shown to play a critical role in ccRCC progression, which is consistent with previous evidence showing that overexpression of PIM1 contributes to the progression of cancers, including breast cancer, mesothelioma and glioblastoma. Conversely, by acting as a suppressor, PIM1 is frequently downregulated in other human malignancies, such as prostate cancer, pancreatic ductal carcinoma and non-small-cell lung cancer; in these cases, PIM1 expression is correlated with a good prognosis^26–28^. These conflicting data surrounding PIM1 function can be partly explained by the different cancer types examined and the different associated signalling pathways, which are involved in a wide variety of processes, including proliferation, cell cycle arrest, apoptosis, migration, invasion and drug resistance.

Furthermore, we showed that depletion of PIM1 effectively blocked EMT through the upregulation of E-cadherin expression and downregulation of N-cadherin and Vimentin expression in ccRCC cells. We further showed that knockdown of PIM1 significantly repressed the expression of EMT-inducing transcription factors (ZEB1, ZEB2, Snail1, Snail2 and Twist). Taken together, our results suggest that depletion of PIM1 attenuates EMT in ccRCC cells by downregulating EMT-promoting transcription factor expression. Additionally, matrix metalloproteinases (MMPs), which are pathologically related to EMT, modify the ECM to enable cell motility and induce the expression of transcription factors associated with EMT to facilitate EMT^29,30^. The increased levels of EMT-associated transcription factors further activate MMP expression, which further upregulates the expression of EMT-inducing transcription factors, thus generating a feed-forward regulatory loop^31^. In this study, we demonstrated that both MMP2 and MMP9 proteins were significantly decreased when EMT was attenuated by the depletion of PIM1, indicating that knockdown of PIM1 potentially impairs the abovementioned MMP2 and MMP9 regulatory loop, thereby blocking EMT.

Next, we found that depletion of PIM1 remarkably decreased the phosphorylation of both Smad2 and Smad3 in ccRCC cell nuclei without significantly changing the total Smad2 and Smad3 protein levels, suggesting that PIM1 regulates Smads via interactions with Smad2 or Smad3 in the nucleus and that the subsequent phosphorylation of Smad2 and Smad3 induces EMT. Additionally, canonical TGF-β signalling pathway initiation upon TGF-β binding to TβRII and TβRI on the cell surface leads to TβRI phosphorylation by TβRII, and activation of TβRI further phosphorylates downstream the transcriptional factors Smad2 and Smad3 at two C-terminal serines in the cytoplasm. The Smad2 and Smad3 proteins then combine with Smad4 to form a trimeric Smad complex that subsequently translocates into the nucleus, where they cooperate with transcriptional co-activators or transcriptional co-repressors to regulate target gene transcription^32,33^. Together with the above signalling transducer model, our data suggest that the PIM1 interaction with Smad2 or Smad3 in the nucleus and the subsequent phosphorylation of Smad2 and Smad3 that triggers EMT may serve as a supplement for the canonical TGF-β-induced signalling pathway.

One important finding reported here is the cooperation of PIM1 with c-Myc to induce EMT in ccRCC. The current results reveal that phosphorylation of c-Myc via interactions with PIM1 activates the expression of transcription factors (ZEB1, ZEB2, Snail1, Snail2 and Twist) to synergistically promote EMT without activating Smads. These observations indicate a potential molecular mechanism in which PIM1 mediates crosstalk between signalling pathways, and independent Smads and c-Myc target downstream transcription factors to trigger EMT. In addition, we found that depletion of PIM1 and the subsequent blockage of signalling pathway crosstalk and EMT is reversed by TGF-β, which activates the canonical TGF-β-induced signalling pathway to enhance the phosphorylation of both Smad2 and Smad3 in the nucleus; this further confirmed the molecular mechanism of PIM1 in our study.

Mutation of the von Hippel–Lindau (VHL) tumour suppressor plays an essential role in the cellular oxygen-sensing pathway, which is correlated with poor prognosis in ccRCC patients^34^. VHL, which is located on chromosome 3p, is mutated in 52% of ccRCCs, and the mutation generally leads to dysfunction^35^. VHL targets hypoxia-inducible factor (HIF) for degradation under normal physiological conditions, whereas in the tumour cell microenvironment, mutation of VHL enables HIF to accumulate in the tumour cells. The elevated HIF levels then facilitate the expression of a series of downstream target genes, including vascular endothelial growth factor (VEGF) and other genes involved in angiogenesis and cancer progression^36^. Remarkably, HIF1α induces EMT and promotes tumour metastasis under hypoxic conditions via upregulation of the Twist gene^37^. Tumour cell growth further promotes the enrichment of HIF1α in tumour cells, which forms a feedback loop in the tumour cell microenvironment. Intriguingly, previous data have demonstrated that HIF1α induces PIM1 expression in solid tumours during hypoxia^38,39^. Moreover, our results showed that depletion of PIM1 strongly impaired angiogenesis and blocked EMT in ccRCC cells. The present data, together with previous evidence, suggest that PIM1 may participate in the VHL and HIF1α feedback loop to induce angiogenesis and EMT in ccRCC. However, the underlying molecular signalling pathways remain to be elucidated in the future.

Multiple studies have shown that the correlation between EMT and cancer stem cell (CSC) properties may help explain cancer progression and dissemination^40–42^. Tumour cell plasticity plays a crucial role in the multistep process of cancer progression towards metastasis, in which epithelial-like tumour cells generate CSCs with both a mesenchymal and stem-cell-like phenotype following dedifferentiation through EMT; these CSCs subsequently enter circulation to form metastases at distant sites^43,44^. Therefore, further investigations are required to determine whether depletion of PIM1 impairs the dynamic phenotypic transition of ccRCC cells.

In conclusion, our study showed that PIM1 is a crucial modulator of human ccRCC progression, migration, invasion and angiogenesis. Additionally, a potential molecular mechanism for PIM1 in the regulation of ccRCC development and progression through mediating crosstalk between signalling pathways involved in EMT, including independent Smads and c-Myc, was identified in our study. These data suggest that PIM1 may be a new therapeutic target for ccRCC patients.

## Materials and methods

### Antibodies and reagents

Antibodies against PIM1, p-Smad2 (S467), p-Smad3 (S423 and S425) and p-c-Myc (S62) were purchased from Abcam (Cambridge, MA, USA). Antibodies against E-cadherin, N-cadherin, Vimentin, ZEB1, ZEB2, Snail1, Snail2 (Slug), Twist, MMP2, MMP9, Smad2, Smad3, c-Myc, PCNA, Ki67 and GAPDH were purchased from Proteintech Group (Chicago, USA). Wright-Giemsa was purchased from Solarbio (Beijing, China). The inhibitors 10058-F4 and SGI-1776 were purchased from MedChem Express (New Jersey, USA). TGF-β was purchased from PeproTech (New Jersey, USA). The Alexa Fluor 488- and 594-conjugated secondary antibodies were purchased from Invitrogen (CA, USA).

### Cell culture

The human ccRCC cell lines (ACHN, 786-O, OS-RC-2 and 769-P) and the immortalised proximal tubule epithelial cell line HK-2 were originally obtained from the Shanghai Institute of Biochemistry and Cell Biology at the Chinese Academy of Sciences (Shanghai, China). All cells were cultured according to the manufacturer’s protocol. The 786-O, 769-P and OS-RC-2 cell lines were cultured in RPMI-1640 medium containing 10% foetal bovine serum, 100 U/ml penicillin and 100 mg/ml streptomycin, and the ACHN cell line was cultured in MEM medium containing 10% foetal bovine serum, 100 U/ml penicillin and 100 mg/ml streptomycin. HK-2 cells were cultured in DMEM containing 10% foetal bovine serum, 100 U/ml penicillin and 100 mg/ml streptomycin. All cell lines were incubated in a humidified atmosphere containing 5% CO_2_ at 37 °C, and cells between the fifth and tenth passages were used in this study.

### Generation of stable cell lines

ACHN and 786-O cell lines that stably expressed PIM1shRNA or control-shRNA were constructed using a lentiviral shRNA technique. Oligonucleotides were constructed in the LV3 lentiviral RNAi vector (GenePharma, China). Puromycin (5 mg/mL) was used to select for stably transfected cells for 4 weeks. The human PIM1shRNA sequence was designed as follows: 5′-GGTGTGTGGAGATATTCCTTT-3′. The control-shRNA sequence was as follows: 5′-TTCTCCGAACGTGTCACGT-3′.

### Edu assays

Cells were seeded onto coverslips in 24-well plates. Cell proliferation was investigated with the Edu kit (RiboBio, China) according to the manufacturer’s protocol^45^. The stained cells were detected by inverted fluorescence microscopy (IX73, Olympus, Japan). The cells that were double labelled with Edu and Hoechst in the nucleus were identified as positive cells.

### Colony formation assay

One thousand cells were seeded onto 6-well plates and incubated for 14 days until colonies were clearly formed. Then, the plate was gently washed and stained with crystal violet. The number of foci containing >50 cells was determined.

### Wound-healing assay

Cells were grown to confluence in 6-well plates and starved overnight. Then, the confluent monolayers were scraped in a straight line with a sterile 1,000-μL plastic pipette tip to create a linear wound. The wells were washed with PBS to remove all cellular debris, and then, 2 mL of complete medium was added to each well. After a 24-h incubation period, photographs of the wounds were taken with a phase contrast microscope (IX70, Olympus, Japan), and the horizontal distance between the sides of the wound was measured.

### Transwell migration assay

Two hundred microlitres of a cell suspension (5 × 10^4^ cells) in serum-free medium was added to the upper compartment of the Transwell migration chambers (BD, USA). The bottom chamber was filled with 500 μL of medium containing 10% foetal bovine serum. After a 24-h incubation period, cells were fixed with 100% methanol and stained with Wright-Giemsa. The number of cells that penetrated the membrane were counted in five random fields.

### Matrigel transwell assay

The upper compartments of the Transwell migration chambers (BD, USA) were precoated with 50 μL of Matrigel at 4 °C overnight. Then, 200 μL of cell suspension (5 × 10^4^ cells) in serum-free medium was added to the upper compartment of the Transwell migration chambers (BD, USA). The bottom chamber was filled with 500 µL of medium containing 10% foetal bovine serum. After a 24-h incubation period, cells were fixed with 100% methanol and stained with Wright-Giemsa. The number of cells that penetrated the Matrigel and membrane were counted in five random fields.

### Capillary tube formation (CTF) assay

Each well of a 96-well plate was precoated with 50 μL of Matrigel (BD, USA) for 30 min at 37 °C. Then, 200-μL aliquots of human vascular endothelial cells (HUVECs) suspended in supernatant collected from either the ACHN cultures (PIM1shRNA/control-shRNA) or 786-O cultures (PIM1shRNA/control-shRNA) were seeded at 3 × 10^4^ HUVECs per well onto 96-well plates for 6–8 h at 37 °C. CTF was examined under a microscope, and images were captured. The total lengths of the tubes formed by HUVECs were measured with ImageJ software.

### Immunofluorescence assay

Cells (3 × 10^5^) treated with or without 40 µM 10058-F4 or 10 ng/mL TGF-β were grown on glass coverslips in 24-well plates for 24 h. Cells were fixed with 4% formaldehyde, washed with PBS and permeabilized with 0.5% Triton X-100. Next, cells were blocked with 5% goat serum for 30 min at 37 °C and stained with the indicated primary antibody at a dilution of 1:100 in 5% BSA in PBS overnight. The cells were incubated in the dark with DAPI, and immunofluorescence (IF) images of the indicated proteins were acquired with an inverted microscope.

### Real-time PCR (qRT-PCR) assay

Cells (5 × 10^5^) were seeded onto 6-well plates. Total RNA was extracted using TRIzol (Invitrogen, USA) followed by isopropanol precipitation and chloroform extraction. cDNA synthesis was performed using the Reverse Transcriptase System (Invitrogen, USA) according to the manufacturer’s protocol. Real-time PCR (qRT-PCR) was performed with the iCycler Real Time System (Bio-Rad Laboratories, Richmond, CA, USA) using the SYBR Premix EX Tag Master mixture kit (TaKaRa, Japan) according to the manufacturer’s instructions. The sequences of the PIM1 primers were 5′-CTGCTCAAGGACACCGTCTACA-3′ (sense strand) and 5′-GATGGTAGCGGATCCACTCTG-3′ (antisense strand). The sequences of the GAPDH primers were 5′-GGACCTGACCTGCCGTCTAG-3′ (sense strand) and 5′-GTAGCCCAGGATGCCCTTGA-3′ (antisense strand). Relative quantification of PIM1 was performed using the 2^−ΔΔCT^ method normalised to the level of GAPDH.

### Immunoblotting assay

Cells were lysed with RIPA buffer containing protease inhibitors, and protein concentrations were determined using the Pierce BCA protein assay kit (Thermo Scientific, Fremont, CA, USA). Equal amounts of proteins were resolved by SDS-PAGE and then transferred to PVDF membranes. Next, the membranes were blocked with 5% non-fat powered milk in TBST for 1 h at room temperature. Then, the membranes were probed with the indicated primary antibodies overnight at 4 °C followed by secondary antibody incubation. Signals were examined using the ECL chemiluminescence reagent on the ChemiDoc System (Bio-Rad, Hercules, CA, USA).

### Co-immunoprecipitation (Co-IP) assay

Cells at 90% confluence were lysed in IP lysis buffer containing protease inhibitors and precleared with protein G-Sepharose beads for 60 min. Lysates were then incubated with the indicated primary antibodies or non-specific immunoglobulin (IgG) in the presence of protein G-Sepharose beads overnight at 4 °C. The beads were washed with 40 μL of ice-cold elution buffer. Then, 30 μL of 5× sample buffer was added to the eluted samples followed by boiling at 95 °C for 5 min. Protein expression analyses of the indicated proteins were performed by western blotting.

### In vitro kinase assay

Assays were performed using the PIM1 Kinase Assay Kit (Cell Signaling Technology, USA) according to the manufacturer’s recommended protocol.

### Xenografts

The athymic nude mice were assigned to the following two groups: PIM1shRNA and NC (control group). Then, 100 μL of an ACHN (PIM1shRNA/control-shRNA) cell suspension containing 1 × 10^6^ cells was subcutaneously injected into the left flanks of the athymic nude mice (SPF grade, 4–5 weeks old, *n* = 5/per group). The tumour sizes were measured weekly. Twenty-eight days later, the mice were killed, and the tumours were removed for further assessment. All protocols involving live mice were approved by the Animal Care and Use Committee of Dalian Medical University.

### Tumour samples and immunohistochemical analysis

A Tissue Microarray (HKid-CRCC150CS-02) of human ccRCCs, including 75 cases of carcinoma tissues and surrounding tissues, was obtained from Shanghai Outdo Biotech (Shanghai, China). From 2008 to 2010, 56 RCC tissues were collected immediately after surgical resection at the First Affiliated Hospital of Dalian Medical University, and 56 RCC patients were included in a survival analysis. Before inclusion into our study, all patients signed an informed consent form. The study was approved by the First Affiliated Hospital of Dalian Medical University (Dalian, China). Tissues were probed using human PIM1 antibodies. Additionally, IHC for PIM1, Ki67 and PCNA in mouse tumours from each group was performed. The edge tissues of the mouse tumours from the PIM1 knockdown and control groups were subjected to IHC for p-Smad2, p-Smad3 and p-c-Myc. The immunostained tissues were scored by multiplying the intensity (0–3) and extent (0–100) of staining, which has been previously described^46^.

### Statistical analysis

Data were expressed as the mean ± S.E.M. of the values from triplicate experiments. Statistical analyses were conducted using SPSS package (version 23). The two-tailed Student’s *t*-test was used to compare two groups of independent samples. One-way ANOVA was used for multiple comparisons. Kaplan–Meier survival analysis was used to demonstrate the prognostic relevance of PIM1 in a univariate analysis. Significant differences were indicated as follows: *p < 0.05; and **p < 0.01.

## Electronic supplementary material

Supplementary Information (Supplementary Figure Legend)

Supplementary Figure 1

Supplementary Figure 2

Supplementary Figure 3
