# Correction to: PIM1 mediates epithelial-mesenchymal transition by targeting Smads and c-Myc in the nucleus and potentiates clear-cell renal-cell carcinoma oncogenesis

**DOI:** 10.1038/s41419-021-03387-3

**Published:** 2021-03-04

**Authors:** Bin Zhao, Lei Liu, Jun Mao, Zhiwei Zhang, Qifei Wang, Quanlin Li

**Affiliations:** 1 Department of Urology, The First Affiliated Hospital of Dalian Medical University, No. 222 Zhongshan Road, Dalian, 116011 China; 2 The Key Laboratory of Tumour Stem Cell Research of Liaoning Province, Dalian Medical University, Dalian, 116044 China

Correction to: *Cell Death and Disease*

10.1038/s41419-018-0348-9 published online 22 February 2018

The original version of this article unfortunately contained a mistake in Fig. 6D. The correct figure can be found below. The authors apologize for the mistake. The original article has been corrected.

**Figure:**
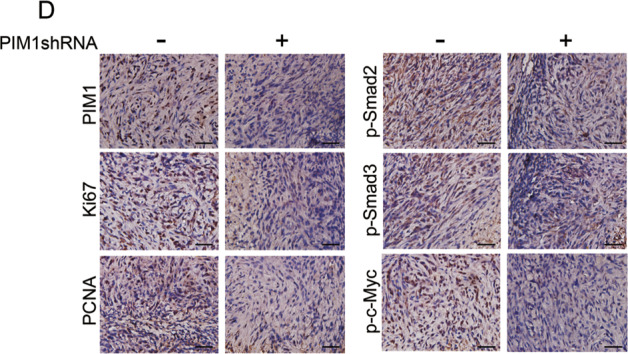
Fig. 6▓.

