# Supplementary material for: PIM1 mediates epithelial-mesenchymal transition by targeting Smads and c-Myc in the nucleus and potentiates clear-cell renal-cell carcinoma oncogenesis
Source: Cell Death Dis. 2018 Feb 22;9(3):307. doi: 10.1038/s41419-018-0348-9 (PMC5833424; doi:10.1038/s41419-018-0348-9)
Supplement: Supplementary file 1 — Supplementary Information (Supplementary Figure Legend) [file 41419_2018_348_MOESM1_ESM.docx]

**Supplementary Figure 1** Direct phosphorylation of Smads by PIM1. A and B. In vitro kinase assay of PIM1. Endogenous PIM1/Smad complexes were immunopurified from ACHN and 786-O cell lysates with a PIM1 antibody and assayed for PIM1 activity towards Smad2 or Smad3 in the presence or absence of SGI-1776 (5 µM, a novel small molecule inhibitor of PIM1 kinase activity). Phosphorylation was detected by immunoblotting using antibodies against p-Smad2 (S467) or p-Smad3 (S423 and S425). All data are from three independent experiments.

**Supplementary Figure 2** Smad2 does not interact with Smad3 in the PIM1/Smad complex. A and B. Co-IP assays. Endogenous PIM1/Smad complexes were immunopurified from ACHN and 786-O cell lysates with a PIM1 antibody. Then, the anti-p-Smad2 (S467) antibody or IgG (control) was used to immunoprecipitate p-Smad2 (S467) from PIM1/Smad complexes. Immunoblotting assays using the anti-p-Smad3 (S423 and S425) antibody were used to detect p-Smad3 (S423 and S425) protein levels. All data are from triplicate experiments.

**Supplementary Figure 3** PIM1 interacts with c-Myc and phosphorylates c-Myc at S62. A and B. Co-IP assays. Endogenous PIM1 was immunoprecipitated, and the eluted proteins were probed for p-c-Myc (S62). Lysates were also subjected to co-IP with an IgG control. All data are from three biological replicates.
