# Supplementary figures and images for: PIM1 mediates epithelial-mesenchymal transition by targeting Smads and c-Myc in the nucleus and potentiates clear-cell renal-cell carcinoma oncogenesis
Source: Cell Death Dis. 2018 Feb 22;9(3):307. doi: 10.1038/s41419-018-0348-9 (PMC5833424; doi:10.1038/s41419-018-0348-9)

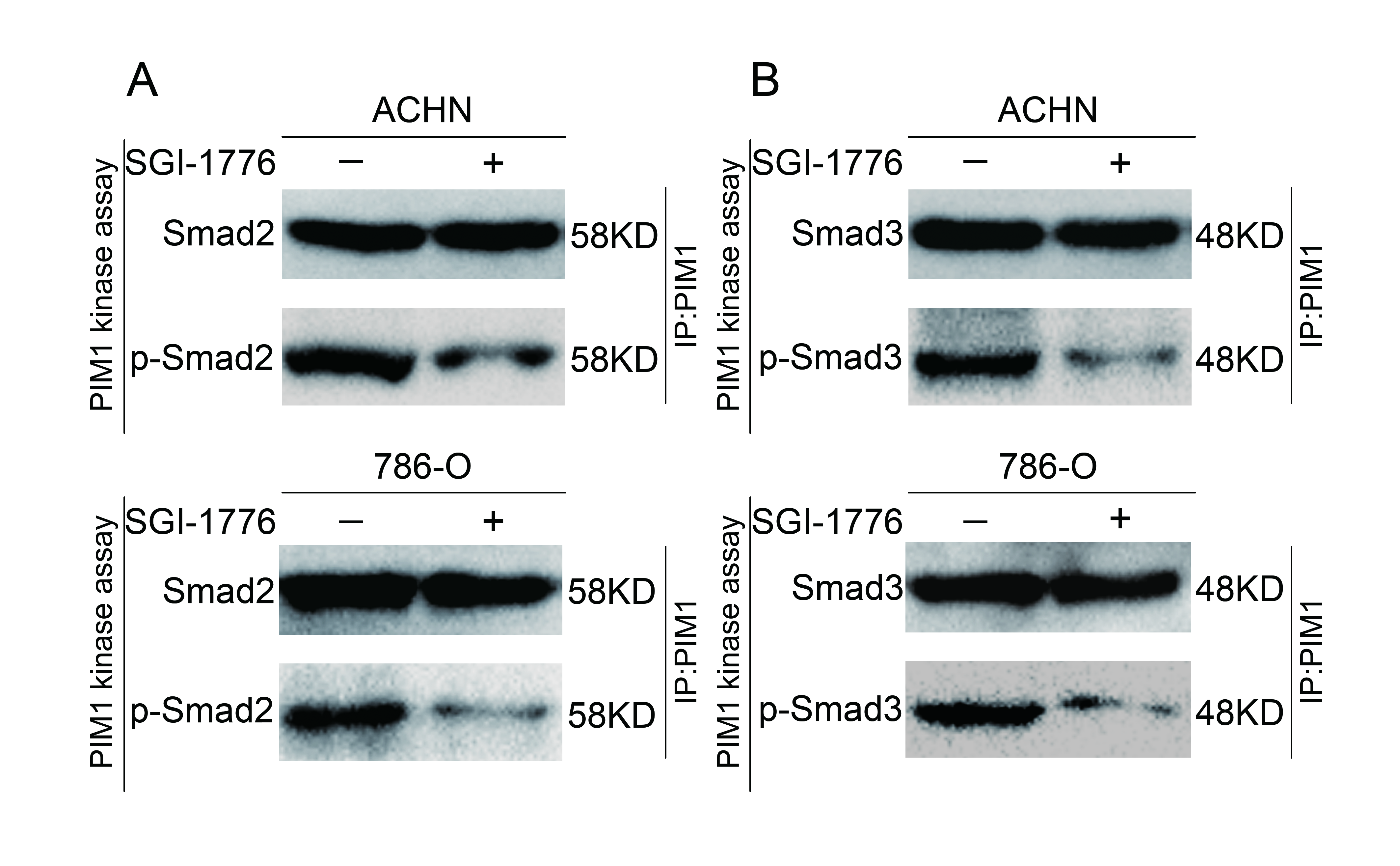

Supplement: Supplementary file 2 — Supplementary Figure 1 [file 41419_2018_348_MOESM2_ESM.tif]

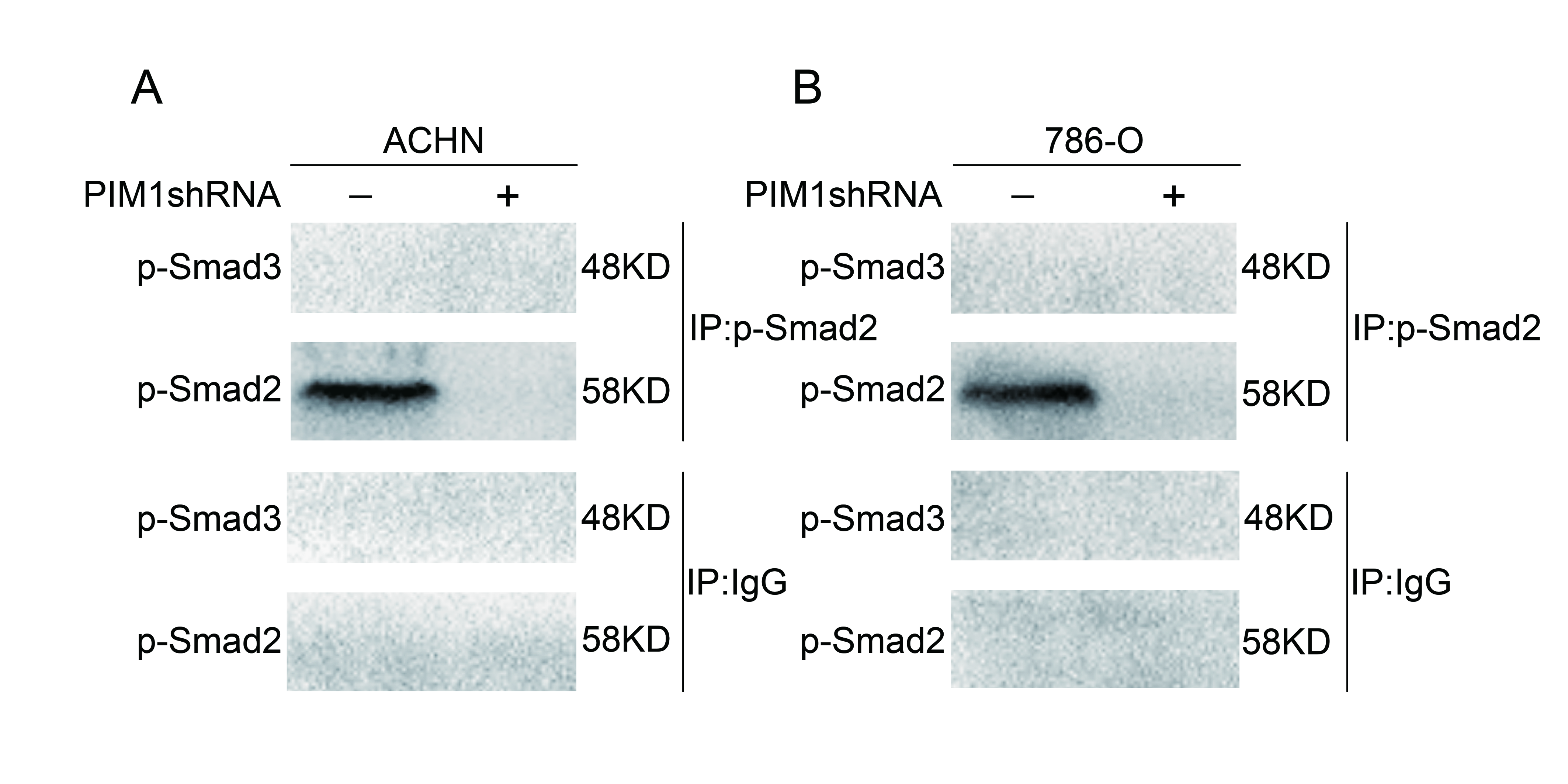

Supplement: Supplementary file 3 — Supplementary Figure 2 [file 41419_2018_348_MOESM3_ESM.tif]

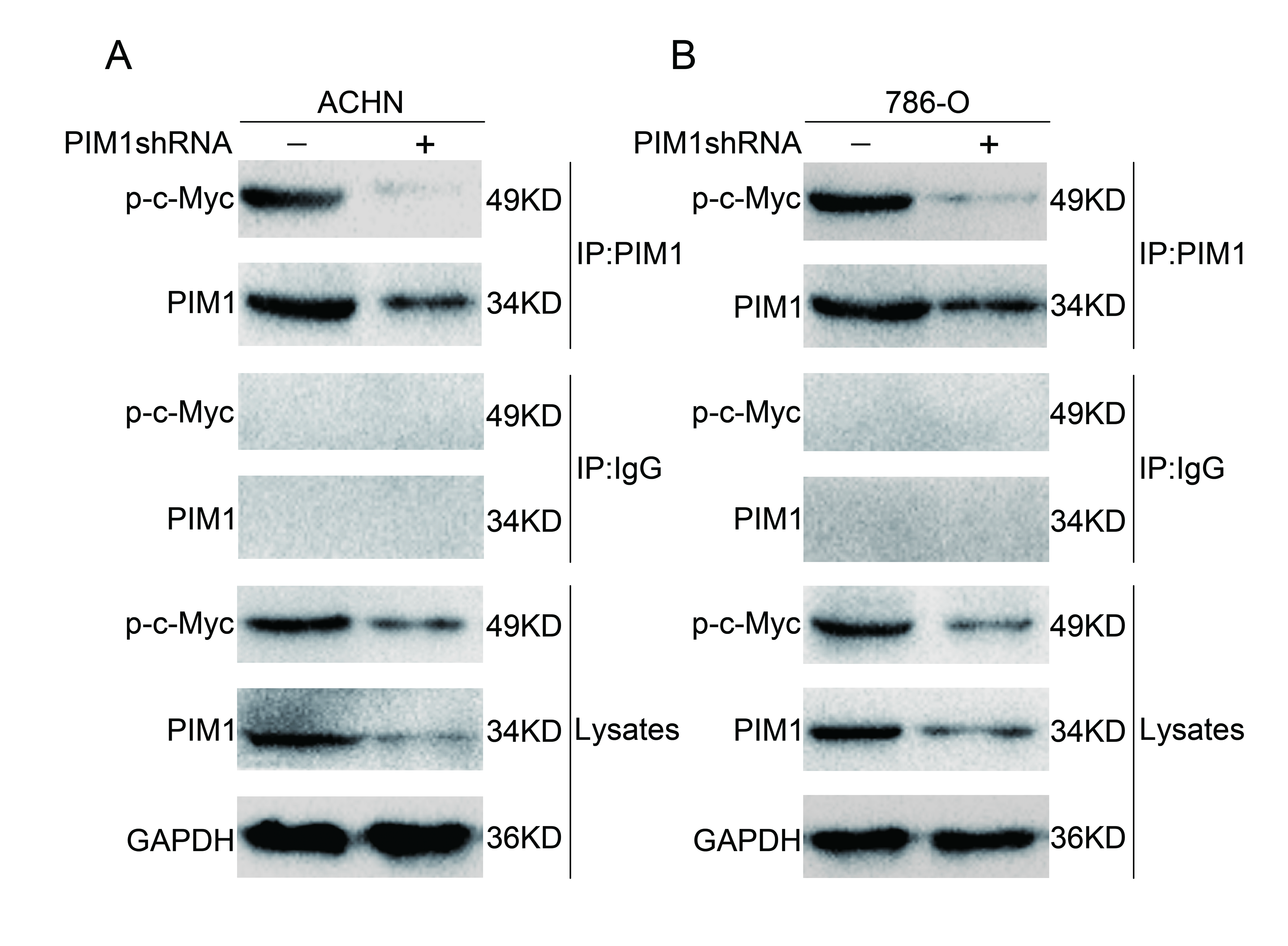

Supplement: Supplementary file 4 — Supplementary Figure 3 [file 41419_2018_348_MOESM4_ESM.tif]
